# Sildenafil, a phosphodiesterase-5 inhibitor, stimulates angiogenesis and bone regeneration in an atrophic non-union model in mice

**DOI:** 10.1186/s12967-023-04441-8

**Published:** 2023-09-08

**Authors:** Maximilian M. Menger, David Bauer, Michelle Bleimehl, Claudia Scheuer, Benedikt J. Braun, Steven C. Herath, Mika F. Rollmann, Michael D. Menger, Matthias W. Laschke, Tina Histing

**Affiliations:** 1 Department of Trauma and Reconstructive Surgery, Eberhard Karls University Tuebingen, BG Trauma Center Tuebingen, 72076 Tuebingen, Germany; 2 Institute for Clinical and Experimental Surgery, Saarland University, 66421 Homburg, Saar Germany

**Keywords:** Non-union, Sildenafil, Segmental defect, Bone regeneration, Fracture healing, Angiogenesis, Mice

## Abstract

Non-union formation represents a major complication in trauma and orthopedic surgery. The phosphodiesterase-5 (PDE-5) inhibitor sildenafil has been shown to exert pro-angiogenic and pro-osteogenic effects in vitro and in vivo. Therefore, the aim of the present study was to analyze the impact of sildenafil in an atrophic non-union model in mice. After creation of a 1.8 mm segmental defect, mice femora were stabilized by pin-clip fixation. Bone regeneration was analyzed by means of X-ray, biomechanics, photoacoustic and micro-computed tomography (µCT) imaging as well as histological, immunohistochemical and Western blot analyses at 2, 5 and 10 weeks after surgery. The animals were treated daily with either 5 mg/kg body weight sildenafil (n = 35) or saline (control; n = 35) per os. Bone formation was markedly improved in defects of sildenafil-treated mice when compared to controls. This was associated with a higher bending stiffness as well as an increased number of CD31-positive microvessels and a higher oxygen saturation within the callus tissue. Moreover, the bone defects of sildenafil-treated animals contained more tartrate-resistant acid phosphatase (TRAP)-positive osteoclasts and CD68-positive macrophages and exhibited a higher expression of the pro-angiogenic and pro-osteogenic markers cysteine rich protein (CYR)61 and vascular endothelial growth factor (VEGF) when compared to controls. These findings demonstrate that sildenafil acts as a potent stimulator of angiogenesis and bone regeneration in atrophic non-unions.

## Introduction

Delayed bone healing and non-union formation remain major complications in trauma and orthopedic surgery. Despite increasing insights into the cellular and molecular mechanisms of bone regeneration, effective treatment strategies are missing and up to 10% of all fractures still fail to heal [1]. Non-unions are commonly defined as failure to achieve union by 9 months since the injury, and for which there has been no signs of healing for 3 months [2, 3]. However, other authors define non-union formation in long bones as no radiological sign for fracture healing after a period of 6 months [4]. Notably, the diagnosis of non-unions should include both the clinical and radiological examination of the patient [5].

In many cases, the treatment of non-unions is highly challenging and requires extensive revision surgery. Accordingly, failed fracture healing is not only associated with a major economic burden for the society, but also results in a significant loss of function and pain for the patient [6]. Excessive interfragmentary movements, infections, associated soft-tissue injury and systemic comorbidities bear a high risk for non-union formation [7, 8]. Non-unions can be differentiated in hypertrophic non-unions, which are generally the result of an insufficient mechanical stability and can be treated in most cases by providing a stable osteosynthesis [3, 9]. Atrophic non-unions, however, are considered to be the result of an avascular and biologically inert environment at the fracture site and lack adequate treatment options, making them a major clinical burden [3, 10]. Moreover, in a considerable number of cases, the reason for the failure of fracture healing remains unclear and, until now, there is no consensus on the ideal management [11]. Accordingly, further research is required to elucidate the pathophysiology of non-union formation and to develop effective treatment strategies.

Bone regeneration is a complex, sequentially orchestrated process, involving the coordination of multiple growth factors and cell types. Within this process sufficient vascularization has been recognized as one of the major prerequisites for successful fracture healing [12]. On the other hand, impaired angiogenesis at the fracture site may lead to delayed healing or even atrophic non-union formation [13].

The enzyme phosphodiesterase-5 (PDE-5) catalyzes the breakdown of cyclic guanosine monophosphate (cGMP), one of the most potent stimulators of smooth muscle relaxation. In recent years, sildenafil, a selective inhibitor of PDE-5, has become the most commonly used drug for the treatment of erectile dysfunction in men due to its potent action of enhancing cGMP accumulation and subsequent vasodilation in the corpus cavernosum [14]. Interestingly, a variety of experimental studies reported that sildenafil also exerts pro-angiogenic effects by the upregulation of pro-angiogenic growth factors [15, 16]. Moreover, we could already demonstrate that sildenafil accelerates fracture healing by upregulating the pro-osteogenic factor cysteine rich protein (CYR)61 [17]. However, it is not known whether sildenafil is also capable of stimulating angiogenesis and bone formation in atrophic non-unions. Accordingly, we herein analyzed for the first time the effects of sildenafil in a non-union model in mice.

## Materials and methods

### Animals

A total number of 70 CD-1 mice with a body weight of 30–40 g and an age of 12–16 weeks was used. The mice were bred at the Institute for Clinical and Experimental Surgery, Saarland University, Germany, and housed at a regular light and dark cycle with free access to tap water and standard pellet food (Altromin, Lage, Germany).

All experiments were performed according to the German legislation on the protection of animals and the National Institutes of Health (NIH) Guide for the Care and Use of Laboratory Animals (Institute of Laboratory Animal Resources, National Research Council, Washington DC, USA). The experiments were approved by the local governmental animal protection committee (permit number: 13/2019).

### Surgical procedure

Mice were anesthetized by an intraperitoneal (i.p.) injection of ketamine (75 mg/kg body weight; Ursotamin®, Serumwerke Bernburg, Bernburg, Germany) and xylazine (15 mg/kg body weight; Rompun®, Bayer, Leverkusen, Germany). To evaluate the effects of sildenafil on bone regeneration, we used a well-established murine non-union model, as introduced by Garcia et al. [18].

Under aseptic conditions, a ~ 4 mm medial parapatellar incision was created at the right knee and the patella was dislocated laterally. After drilling a hole (diameter of 0.5 mm) into the intracondylar notch, a distally flattened press fit 24 Gauge needle (diameter of 0.55 mm) was implanted intramedullary and the wound was closed. The pin was flattened at the distal end to avoid secondary dislocation. After insertion of the pin, the diaphysis of the femur was exposed by a lateral approach. Subsequently, a custom-made clip of 6 mm length was implanted ventrodorsally into the femur and lateral of the already implanted pin. A gap size of 1.8 mm was created by means of a spherical trephine under permanent saline solution cooling. Moreover, the periosteum was stripped 2 mm proximally and distally of the gap along the longitudinal axis of the femoral bone. The implant position was confirmed by radiography (MX-20, Faxitron X-ray Corporation, Wheelin, IL, USA). All procedures were done under an operating microscope, guaranteeing a high level of precision. For analgesia the mice received tramadol-hydrochloride (Grünenthal, Aachen, Germany) in the drinking water 1 day prior to surgery until 3 days after surgery.

Thirty-five mice were treated daily with 5 mg/kg body weight sildenafil (Viagra®, Pfizer, Germany; dissolved in saline) per os (p.o.) by gavage. Control animals (n = 35) received equal amounts of saline. At 2 weeks (n = 8 each group), 5 weeks (n = 8 each group) and 10 weeks (n = 10 each group) the animals were euthanized by an overdose of anesthetics and the femora were excised for further radiological and histological analyses. Additional animals were euthanized accordingly at 2 weeks (n = 3 each group), 5 weeks (n = 3 each group) and 10 weeks (n = 3 each group) for Western blot analyses of the callus tissue.

### X-ray analysis

At 2, 5 and 10 weeks after surgery the animals were anesthetized and ventrodorsal radiographs of the osteotomized femora were performed. The process of bone regeneration was analyzed according to the Goldberg score with stage 0 indicating radiological non-union, stage 1 indicating possible union and stage 2 indicating radiological union [19].

### Biomechanical analysis

After removal of the soft tissue and the implants, the bending stiffness of the isolated femora was measured by a 3-point-bending device using a non-destructive approach. This allowed the subsequent use of the specimens for micro computed tomography (µCT), histology and immunohistochemistry and, thus, a reduction of the number of required laboratory animals. Due to the different stages of healing, the loads, which had to be applied, markedly varied between the individual animals and time points. Loading was stopped individually in every case when the actual load-displacement curve deviated more than 1% from linearity. Bending stiffness (N/mm) was calculated from the linear elastic part of the load-displacement diagram [20].

### Ultrasound and photoacoustic imaging

Ultrasound and photoacoustic imaging were performed at 2, 5 and 10 weeks after surgery by means of a Vevo LAZR system (FUJIFILM VisualSonics Inc.; Toronto, ON, Canada) and a real-time microvisualization LZ550 linear-array transducer (FUJIFILM VisualSonics Inc.) with a center frequency of 40 MHz. For this purpose, the animals were anesthetized with 1.5% isoflurane and subsequently fixed in prone position on a heated stage. The heart rate and respiratory rate were constantly monitored and the body temperature was maintained at 36–37 °C (THM100; Indus Instruments, Houston, TX, USA). A compress was positioned underneath the right hind limb to avoid artefact signals from the heated stage. The skin was shaved dorsally around the right thigh and sterile ultrasound gel was applied to avoid air interference with ultrasound coupling into the animal.

For three-dimensional, high-resolution B-mode ultrasound and OxyHemo-mode photoacoustic image acquisition, the scan head was driven by a linear motor to acquire two-dimensional parallel images at regular spatial intervals of 50 μm over the entire femur. A pulsed laser induced a thermoelastic expansion at the extinction wavelength of 750 nm for Hb and at 850 nm for HbO_2_. The resulting acoustic emissions were detected by a transducer. OxyHemo-mode photoacoustic images were recorded at 750 and 850 nm with a two-dimensional gain of 42 dB. To measure the total hemoglobin (HbT)/volume (1/mm³) within the callus tissue, all detected signals at the two wavelengths were divided by the volume of the callus [21]. Furthermore, oxygen saturation (sO_2_, %) within the callus tissue of unions and non-unions was evaluated, as previously described [22–24]. All values were computed using the Vevo LAB 1.7.2. software (FUJIFILM VisualSonics Inc.).

### µCT analysis

The specimens were scanned (Skyscan 1172, Bruker, Billerica, MA) at a spatial resolution of 6.5 μm with a standardized setup (tube voltage: 50 kV; current: 200 µA; intervals: 0.4°; exposure time: 3500 ms; filter: 0.5 mm aluminum). Images were stored in three-dimensional arrays. To express gray values as mineral content (bone mineral density; BMD), calcium hydroxyapatite (CaHA) phantom rods with known BMD values (0.250 and 0.750 g CaHA/cm^3^) were employed for calibration. The region of interest (ROI) defining the novel bone was contoured manually excluding any original cortical bone. The thresholding allowed the differentiation between poorly and highly mineralized bone. The thresholds to distinguish between poorly and highly mineralized bone were based upon visual inspection of the images, qualitative comparison with histological sections and other studies investigating bone repair and callus tissue by µCT [25, 26]. A BMD with more than 0.642 g/cm^3^, resulting in gray values of 98–255, was defined as highly mineralized bone. Poorly mineralized bone was assumed to have a BMD value between 0.410 g/cm^3^ and 0.642 g/cm^3^, resulting in gray values of 68–97.

The following parameters were calculated from the callus region of interest for each specimen: poorly mineralized bone volume (PM), highly mineralized bone volume (HM), bone volume fraction of tissue volume (BV/TV), bone surface density, trabecular thickness, trabecular separation and trabecular number.

### Histology and histomorphometry

After biomechanical testing and µCT analysis, bones were fixed in paraformaldehyde for 24 h. Subsequently, the specimens were embedded in a 30% sucrose solution for another 24 h and then freezed at − 80 °C. Longitudinal sections through the femoral axis with a thickness of 4 μm were cut by the Kawamotos film method for histomorphometric analyses and stained with Safranin-O. At a magnification of 12.5× (Olympus BX60 Microscope, Olympus, Shinjuku, Japan; Zeiss Axio Cam and Axio Vision 3.1, Zeiss) structural indices were calculated according to the recommendations of Gerstenfeld et al. [27]. The following histomorphometric parameters of the bone defects were evaluated: (i) total callus area, (ii) bone callus area, (iii) cartilaginous callus area and (iv) fibrous callus area. The total callus area was defined as the entire osseous, cartilaginous and fibrous callus tissue between the two drilling holes of the clip outside of the cortices. Pre-existing cortical bone of the proximal and distal fragment, however, was excluded. Each area was marked and calculated using the ImageJ analysis system (NIH, Bethesda, USA).

In addition, tartrate-resistant acid phosphate (TRAP) activity was analyzed in the callus tissue at 2, 5 and 10 weeks after surgery. For this purpose, longitudinal sections of 4 μm were incubated in a mixture of 5 mg naphotol AS-MX phosphate and 11 mg fast red TR salt in 10 mL 0.2 M sodium acetate buffer (pH 5.0) for 1 h at 37 °C. Sections were counterstained with methyl green and covered with glycerin gelatin. TRAP-positive multinucleated cells (three or more nuclei each cell) were counted. In the specimens, one high-power field (HPF, 400× magnification) was placed in a standardized manner in the central region of the callus, while three additional HPFs were placed on each site of the periosteal callus.

### Immunohistochemistry

To analyze the cellular composition within the callus tissue of atrophic non-unions at 2, 5 and 10 weeks after surgery, longitudinal sections with a thickness of 4 μm were cut. For the immunohistochemical detection of microvessels, sections were stained with a monoclonal rat anti-mouse antibody against the endothelial cell marker CD31 (1:100; Abcam, Cambridge, UK). A goat anti-rat IgG-Alexa555 antibody served as secondary antibody (1:100; Life Technology, Eugene, USA). Cell nuclei were stained with Hoechst 33,342 (2 µg/mL; Sigma-Aldrich, Taufkirchen Germany). To detect the neutrophilic granulocyte marker myeloperoxidase (MPO) and the macrophage marker CD68, sections were stained with a polyclonal rabbit anti-mouse antibody against MPO (1:100; Abcam) and a polyclonal rabbit anti-mouse antibody against CD68 (1:100; Abcam). A goat anti-rabbit IgG-antibody (1:200; Dianova, Hamburg, Germany) served as corresponding secondary antibody.

In the specimens, the number of CD31-positive microvessels and the number of MPO- and CD68-positive cells were counted. For this purpose, one HPF was placed in a standardized manner in the central region of the callus, while three additional HPFs were placed on each site of the periosteal callus.

### Western blot analysis

Protein expression within the callus tissue was determined by Western blot analysis, including the expression of CYR61, VEGF, heme oxygenase-1 (HO-1), proliferating cell nuclear antigen (PCNA), receptor activator of NF-κB ligand (RANKL) and osteoprotegerin (OPG). The callus tissue was frozen and stored at − 80 °C until required. Analyses were performed from callus tissue at 2, 5 and 10 weeks after surgery (n = 3 each group). After saving the whole protein fraction, the analysis was performed using the following monoclonal antibodies: sheep anti-mouse CYR 61 (1:300, R&D Systems, Minneapolis, USA), rabbit anti-mouse VEGF (1:300, Abcam, Cambridge, UK), rabbit anti-mouse HO-1 (1:300; Enzo Life Science, Lörrach, Germany), mouse anti-mouse PCNA (1:500, Proteintech Germany GmbH, Planegg-Martinsried, Germany), rabbit anti-mouse RANKL (1:300, Abcam) and rabbit anti-mouse OPG (1:300, Biozol Diagnostica, Eching, Germany). Primary antibodies were followed by corresponding horseradish peroxidase-conjugated secondary antibodies (1:1000, R&D Systems). Protein expression was visualized by means of luminol-enhanced chemiluminescence after exposure of the membrane to the Intas ECL Chemocam Imager (Intas Science Imaging Instrument GmbH, Göttingen, Germany) and normalized to β-actin signals (1:1000, mouse anti-mouse β-actin, Santa Cruz Biotechnology, Heidelberg, Germany) to correct for unequal loading.

### Statistical analysis

All data are given as means ± SEM. After testing the data for normal distribution (Kolmogorov–Smirnov test) and equal variance (*F*-test), comparisons between the two groups were performed by the unpaired Student’s *t*‐test. For non‐parametrical data, a Mann–Whitney *U*‐test was used. All statistics were performed using the SigmaPlot 13.0 software (Jandel Corporation, San Rafael, CA, USA). A p‐value of < 0.05 was considered to indicate significant differences.

## Results

### X-ray analysis

**Fig. 1:**
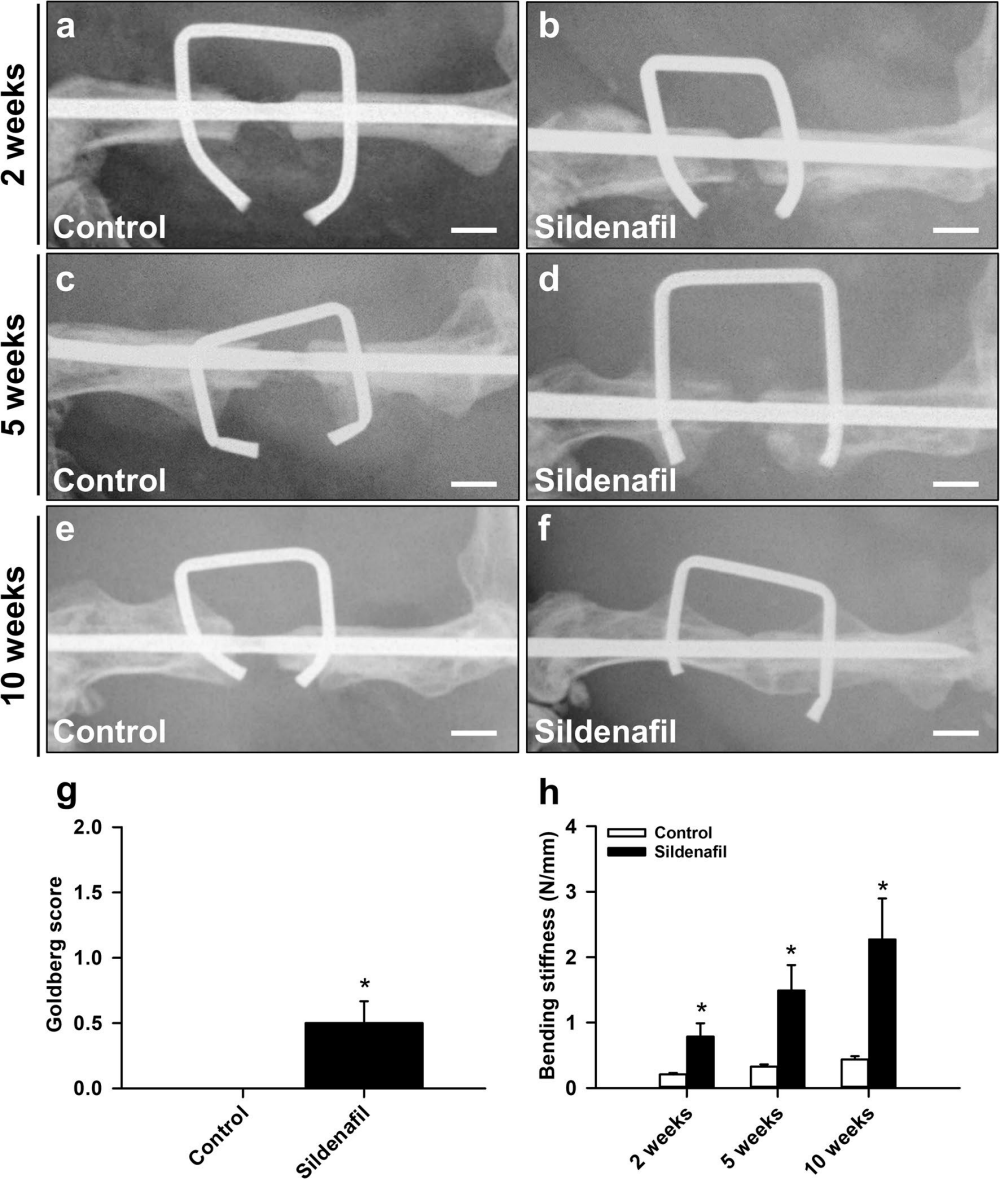
**a**–**f** Representative X-rays of femora of controls and sildenafil-treated mice at 2 (**a**, **b**), 5 (**c**, **d**) and 10 weeks (**e**, **f**) after surgery. Scale bars: 1 mm. **g** Goldberg score of controls (white bar, n = 10) and sildenafil-treated animals (black bar, n = 10) at 10 weeks after surgery, as assessed by X-ray analysis. **h** Bending stiffness (N/mm) of controls (white bars, n = 8 at 2 and 5 weeks, n = 10 at 10 weeks) and sildenafil-treated animals (black bars, n = 8 at 2 and 5 weeks, n = 10 at 10 weeks) at 2, 5 and 10 weeks after surgery, as assessed by biomechanical analysis. Mean ± SEM; *p < 0.05 vs. control

The radiographic analysis revealed a complete lack of osseous bridging of the bone defects in control mice at 2, 5 and 10 weeks after surgery (Fig. 1a, c, e). Accordingly, X-rays demonstrated a reliable non-union formation in all control animals, as indicated by a large persisting gap between the adjoining bone fragments (Fig. 1e). In sildenafil-treated mice the osteotomy gap was also still visible at 2 and 5 weeks after surgery (Fig. 1b, d). However, 10 weeks after surgery we observed osseous bridging in 5 out of 10 sildenafil-treated animals (Fig. 1f). This resulted in a significantly higher Goldberg score at 10 weeks after surgery in sildenafil-treated mice when compared to controls (Fig. 1g).

### Biomechanical analysis

The biomechanical analysis revealed a significantly increased bending stiffness at 2, 5 and 10 weeks after surgery in sildenafil-treated animals when compared to controls (Fig. 1h). Notably, the bending stiffness in control mice remained < 1 N/mm. This indicates failed bone regeneration and non-union formation.

### Ultrasound and photoacoustic imaging

**Fig. 2:**
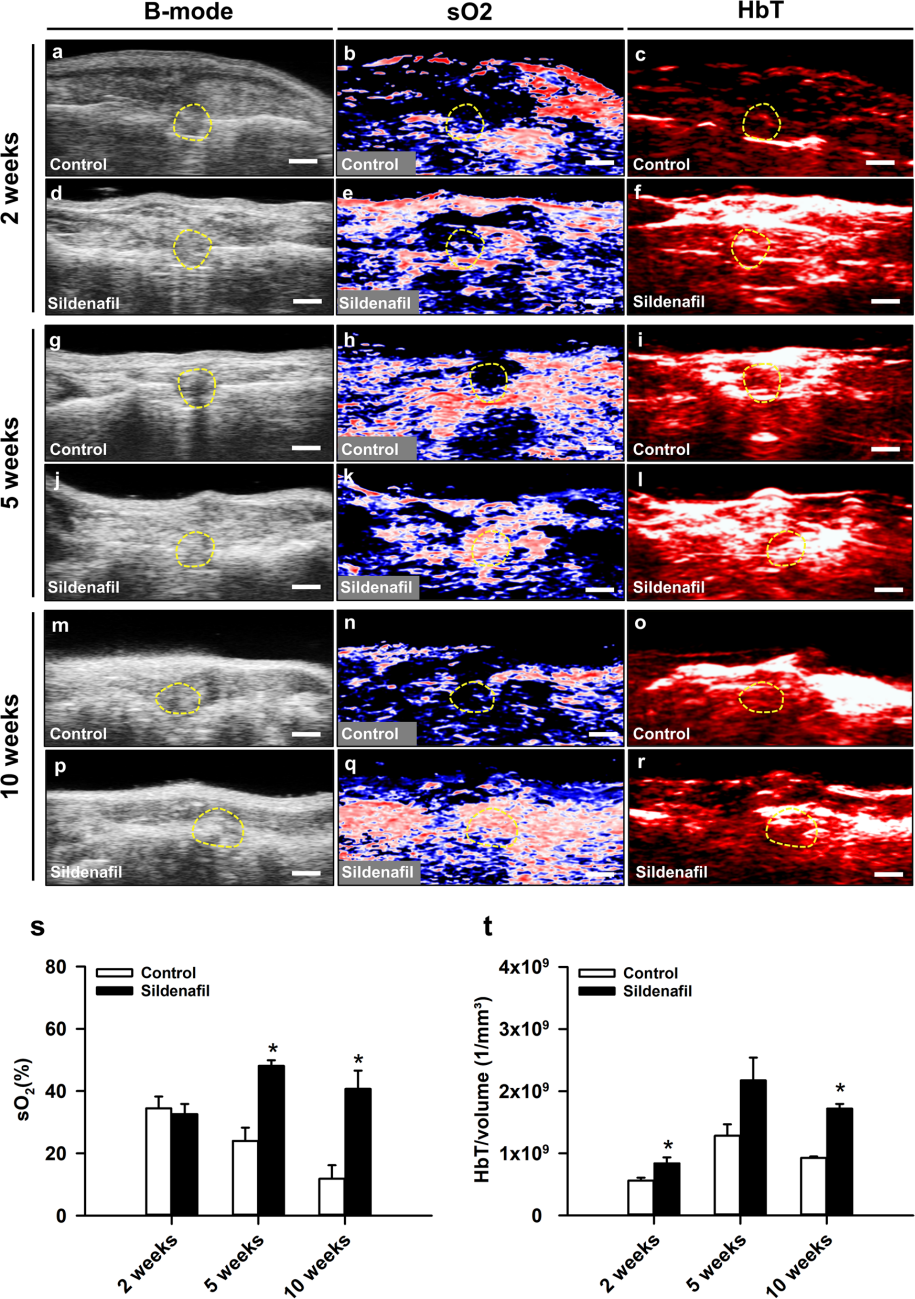
**a**–**r** Representative images of B-mode, hemoglobin oxygen saturation (sO_2_) and total hemoglobin (HbT) in controls and sildenafil-treated animals at 2 (**a**–**f**), 5 (**g**–**l**) and 10 weeks (**m**–**r**) after surgery. The callus tissue is marked by yellow broken lines. Scale bars:1.5 mm. **s** sO_2_ (%) within the callus tissue of controls (white bars, n = 5 at 2, 5 and 10 weeks) and sildenafil-treated animals (black bars, n = 5 at 2, 5 and 10 weeks). **t** HbT/volume (1/mm³) within the callus tissue of controls (white bars, n = 5 at 2, 5 and 10 weeks) and sildenafil-treated animals (black bars, n = 5 at 2, 5 and 10 weeks). Mean ± SEM; *p < 0.05 vs. control

Controls and sildenafil-treated animals were investigated 2 (Fig. 2a–f), 5 (Fig. 2g–l) and 10 weeks (Fig. 2m–r) after surgery by ultrasound and photoacoustic imaging. Ultrasound imaging enabled a clear and reliable visualization of the mouse femur. In control animals, the ultrasound imaging showed a persisting osteotomy gap throughout the entire observation period (Fig. 2a, g, m), whereas callus formation was clearly visible in sildenafil-treated mice 10 weeks after surgery (Fig. 2p). The photoacoustic analysis did not show any difference in sO_2_ within the callus tissue between the two study groups 2 weeks after surgery (Fig. 2s). In contrast, we detected a significantly increased sO_2_ within the callus tissue at 5 and 10 weeks after surgery in sildenafil-treated mice when compared to controls (Fig. 2s). Moreover, we measured a higher amount of HbT/volume, which was found significant at 2 and 10 weeks but not at 5 weeks when compared to controls (Fig. 2t).

### µCT analysis

**Fig. 3:**
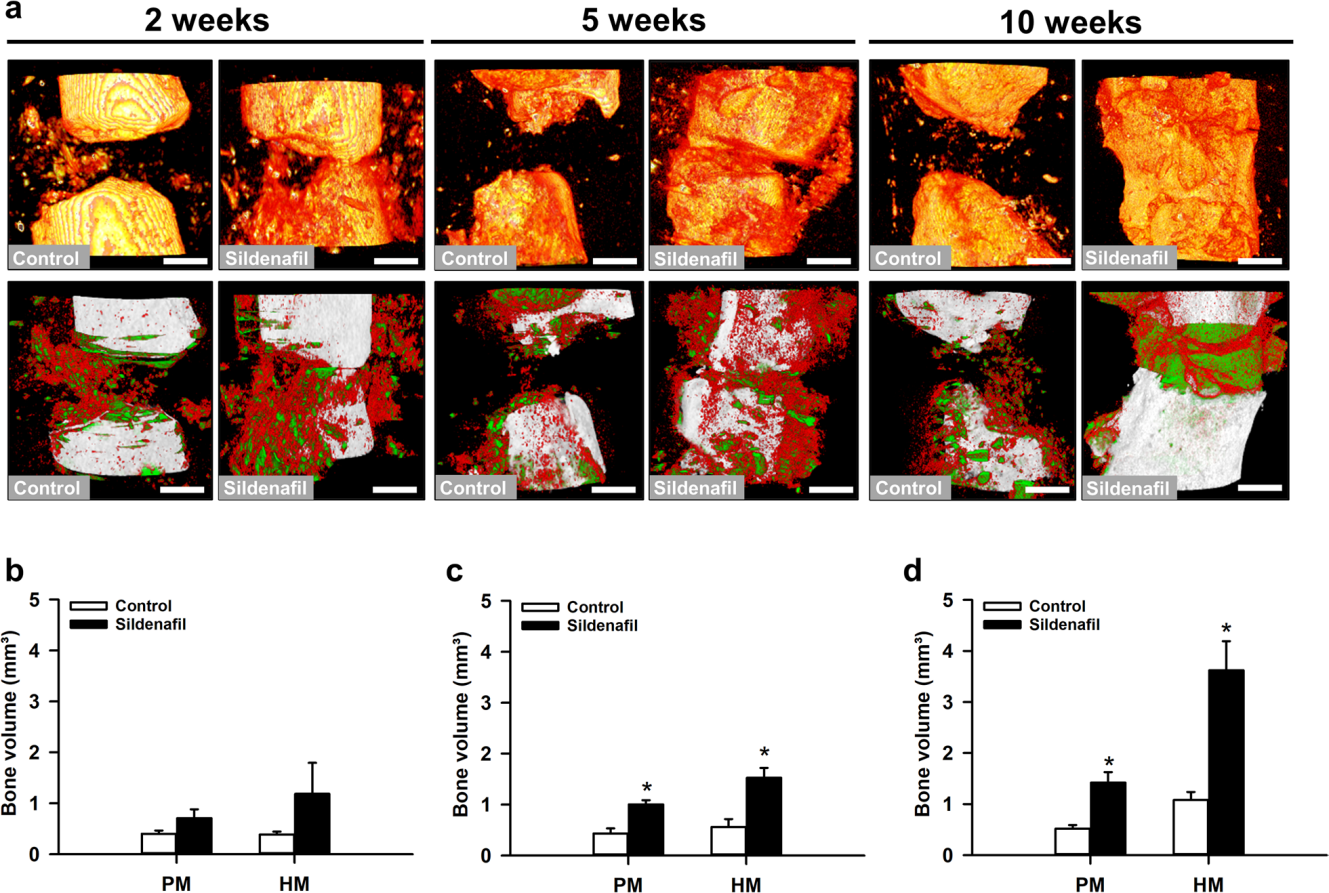
**a** Representative µCT-3D reconstructions of femora in controls and sildenafil-treated mice at 2, 5 and 10 weeks after surgery (yellow: total bone tissue within the upper panel; white: cortical bone, green: poorly mineralized bone, red: highly mineralized bone within the lower panel). Scale bar: 0.5 mm. **b**–**d** Poorly (PM) and highly mineralized (HM) bone volume (mm³) of the callus tissue of controls (white bars, n = 8 at 2 and 5 weeks, n = 10 at 10 weeks) and sildenafil-treated mice (black bars, n = 8 at 2 and 5 weeks, n = 10 at 10 weeks) at 2 (**b**), 5 (**c**) and 10 (**d**) weeks after surgery, as assessed by µCT analysis. Mean ± SEM; *p < 0.05 vs. control

In line with the radiographic results, µCT analyses in the control group revealed a complete lack of osseous bridging throughout the entire observation period, indicating a reliable non-union formation (Fig. 3a). In contrast, signs of osseous bridging were clearly visible in sildenafil-treated animals 10 weeks after surgery (Fig. 3a). Furthermore, we detected a slight increase of poorly and highly mineralized bone tissue volume 2 weeks after surgery under sildenafil treatment, which was even more pronounced at later observation time points (Fig. 3b–d).

**Fig. 4:**
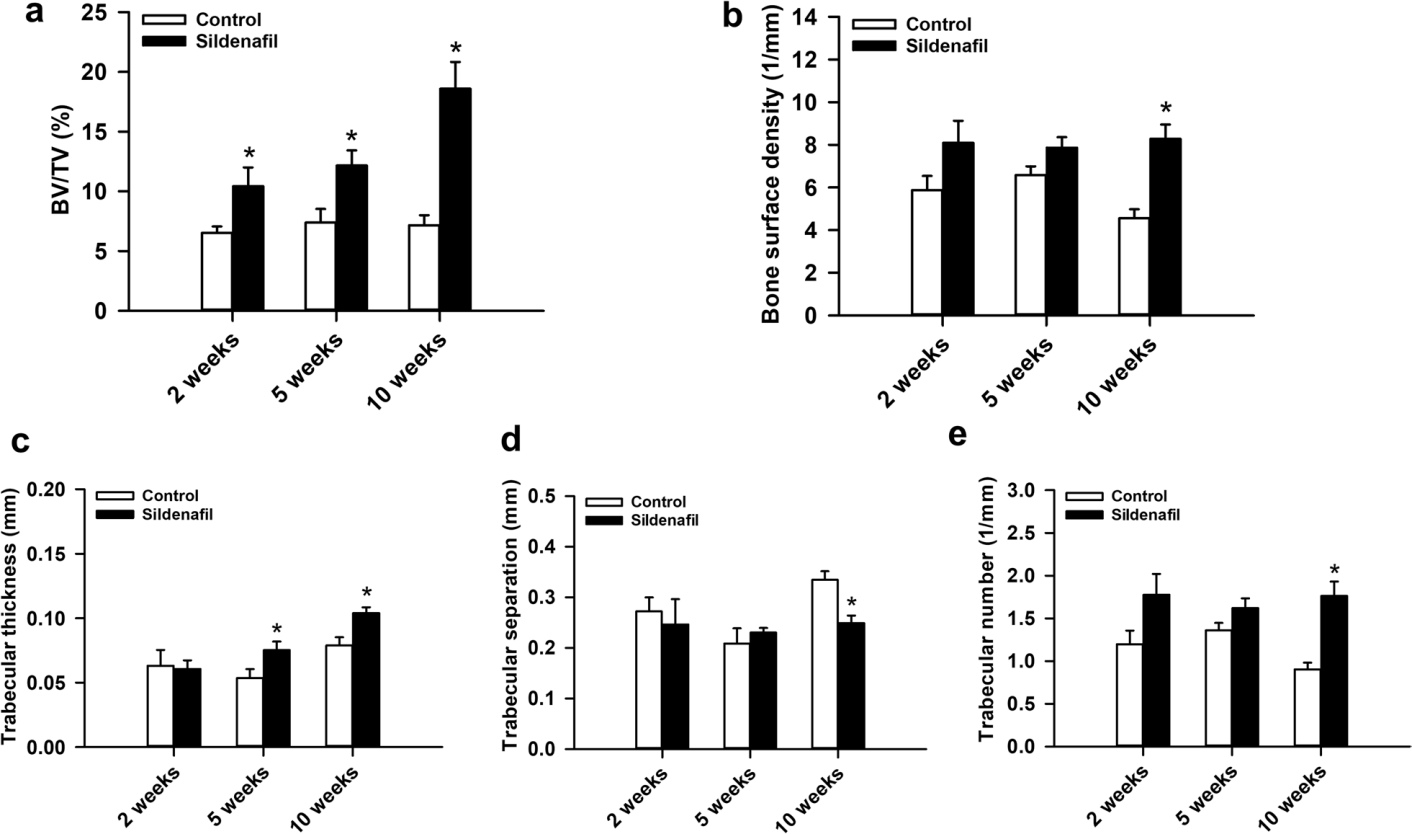
**a**–**e** BV/TV (%) (**a**), bone surface density (1/mm) (**b**), trabecular thickness (mm) (**c**), trabecular separation (mm) (**d**) and trabecular number (1/mm) (**e**) of the callus tissue of controls (white bars, n = 8 at 2 and 5 weeks, n = 10 at 10 weeks) and sildenafil-treated mice (black bars, n = 8 at 2 and 5 weeks, n = 10 at 10 weeks) at 2, 5 and 10 weeks after surgery, as assessed by µCT analysis. Mean ± SEM; *p < 0.05 vs. control

Additional µCT analyses showed a significantly higher BV/TV in sildenafil-treated animals at 2, 5 and 10 weeks after surgery when compared to controls (Fig. 4a). Moreover, the bone surface density was enhanced in the sildenafil group at 10 weeks after surgery (Fig. 4b). Additional investigation of the trabecular architecture demonstrated a higher trabecular thickness at 5 and 10 weeks after surgery under sildenafil treatment (Fig. 4c). Moreover, we observed a lower trabecular separation, and, accordingly, a higher trabecular number in sildenafil-treated mice 10 weeks after surgery when compared to controls (Fig. 4d, e).

### Histomorphometric and histological analysis

**Fig. 5:**
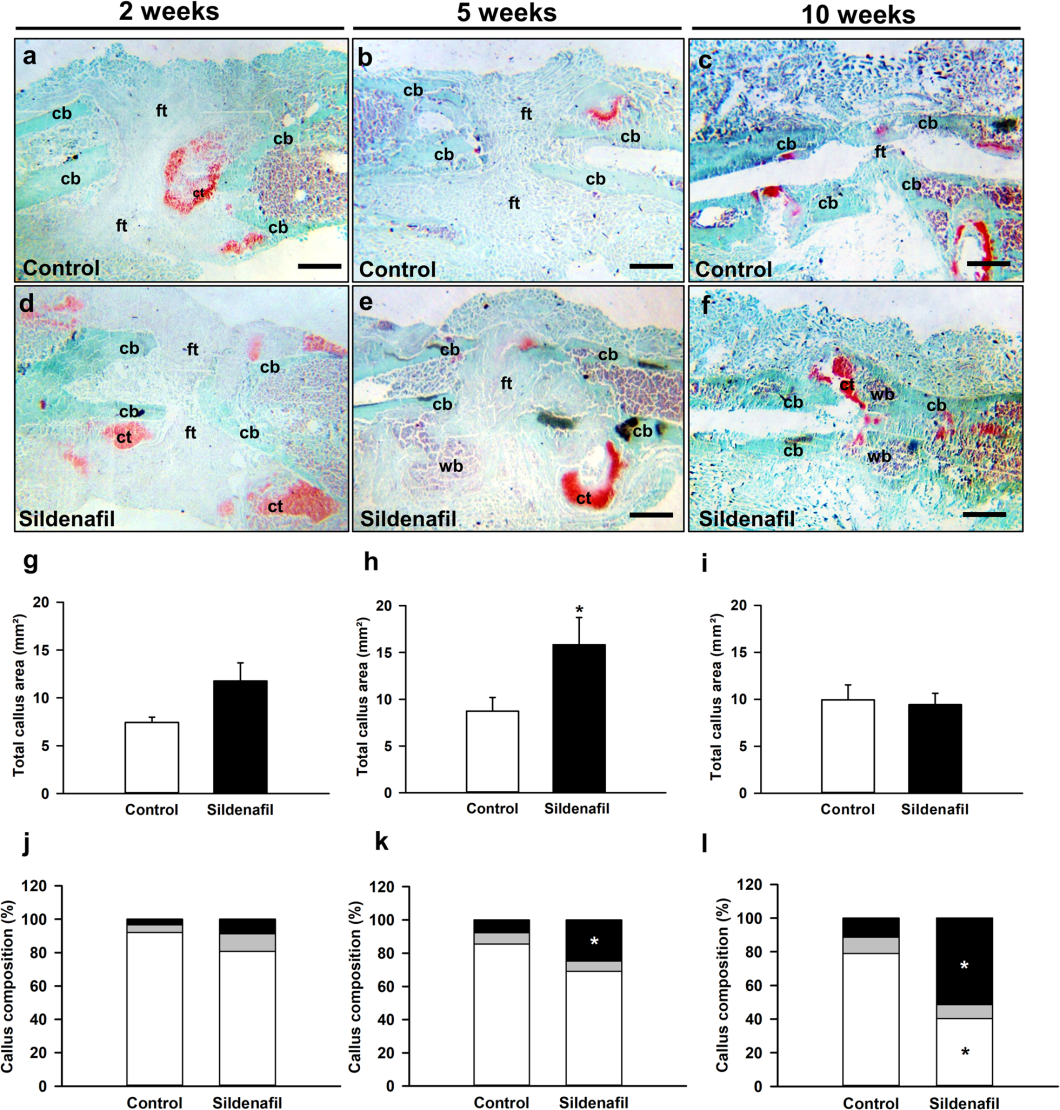
**a**–**f** Representative histological images of Safranin-O-stained femora in controls (**a**–**c**) and sildenafil-treated mice (**d**–**f**) at 2 (**a**, **d**), 5 (**b**, **e**) and 10 weeks (**c**, **f**) after surgery. Fibrous tissue (ft), cartilaginous tissue (ct), woven bone (wb) and cortical bone (cb) are indicated. Scale bars: 0.5 mm. **g**–**i** Total callus area (mm²) of femora of controls (white bars, n = 8 at 2 and 5 weeks, n = 10 at 10 weeks) and sildenafil-treated mice (black bars, n = 8 at 2 and 5 weeks, n = 10 at 10 weeks) at 2 (**g**), 5 (**h**) and 10 (**i**) weeks after surgery. **j**–**l** Callus composition (%), including fibrous tissue (white), cartilaginous tissue (gray) and osseous tissue (black), of femora of controls (n = 8 at 2 and 5 weeks, n = 10 at 10 weeks) and sildenafil-treated mice (n = 8 at 2 and 5 weeks, n = 10 at 10 weeks) at 2 (**j**), 5 (**k**) and 10 (**l**) weeks after surgery. Mean ± SEM; *p < 0.05 vs. control

The histomorphometric analysis demonstrated a lack of osseous bridging in control animals throughout the observation period with abundant fibrous tissue in the osteotomy gap (Fig. 5a–c). In contrast, in sildenafil-treated animals we observed signs of endochondral bone healing at 2 and 5 weeks after surgery and signs of osseous bridging 10 weeks after surgery (Fig. 5d–f). Accordingly, quantitative analyses revealed a significantly larger total callus area at 5 weeks after surgery (Fig. 5h), whereas the total callus area did not significantly differ between the two study groups at 2 and 10 weeks after surgery (Fig. 5g, i). More detailed analyses of the callus composition showed a slightly higher fraction of osseous tissue 2 weeks after surgery but a significantly higher fraction of osseous tissue 5 and 10 weeks after surgery in sildenafil-treated mice when compared to controls (Fig. 5j–l). Accordingly, the fraction of fibrous tissue was slightly lower at 2 and 5 weeks and significantly lower at 10 weeks after surgery under sildenafil treatment (Fig. 5j–l). Notably, the fraction of cartilaginous tissue did not differ between the two study groups throughout the entire observation period (Fig. 5j–l).

**Fig. 6:**
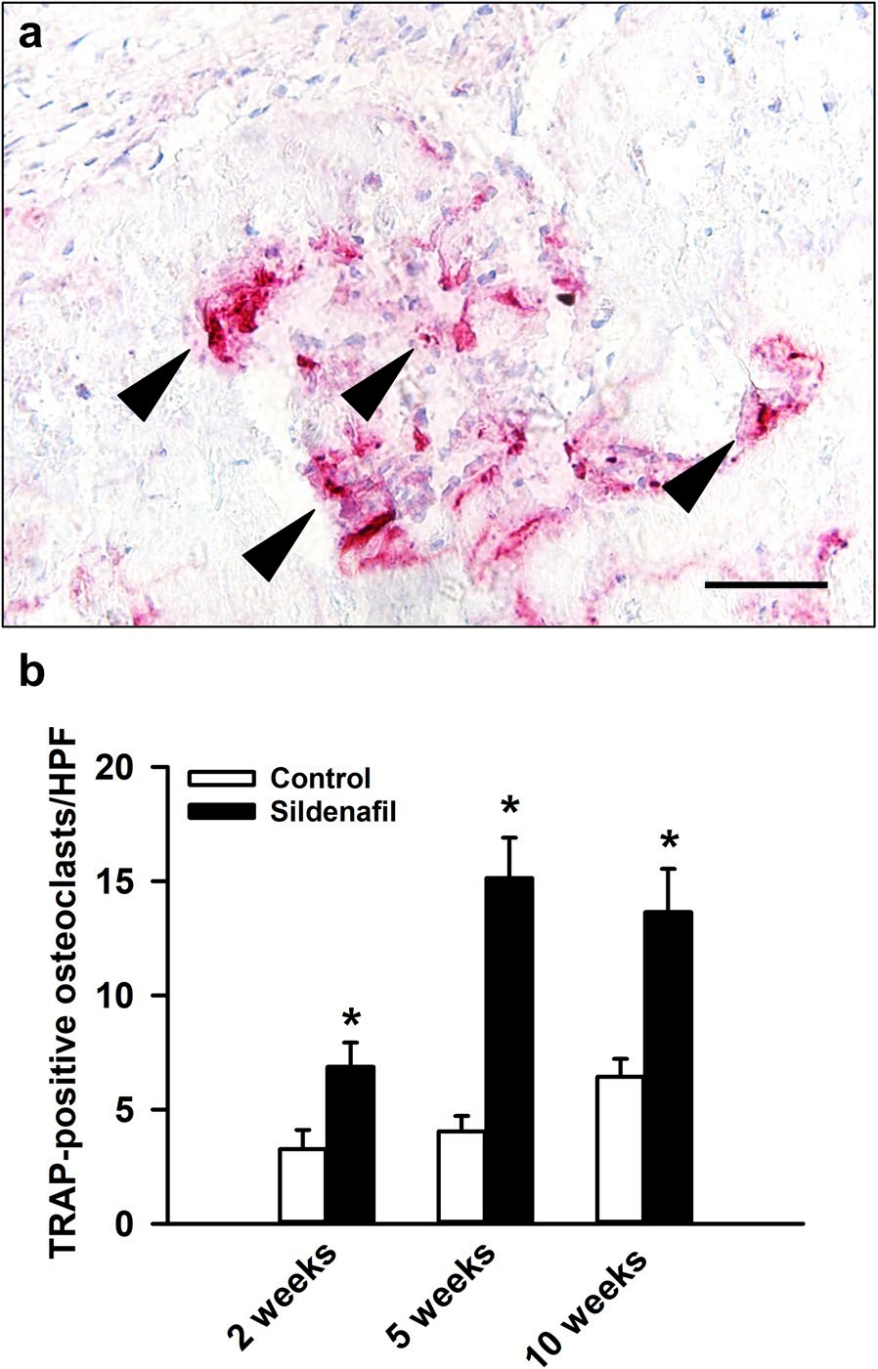
**a** Representative histological image of TRAP-positive osteoclasts (arrows) within the callus tissue of a control mouse at 2 weeks after surgery. Scale bar: 20 μm. **b** TRAP-positive osteoclasts/HPF within the callus tissue of controls (white bars, n = 8 at 2 and 5 weeks, n = 10 at 10 weeks) and sildenafil-treated mice (black bars, n = 8 at 2 and 5 weeks, n = 10 at 10 weeks) at 2, 5 and 10 weeks after surgery, as assessed by histological analysis. Mean ± SEM; *p < 0.05 vs. control

In addition, we analyzed the number of TRAP-positive osteoclasts within the callus tissue. This analysis revealed a significantly higher number of TRAP-positive osteoclasts in sildenafil-treated mice at 2, 5 and 10 weeks after surgery when compared to controls (Fig. 6a, b).

### Immunohistochemical analysis

**Fig. 7:**
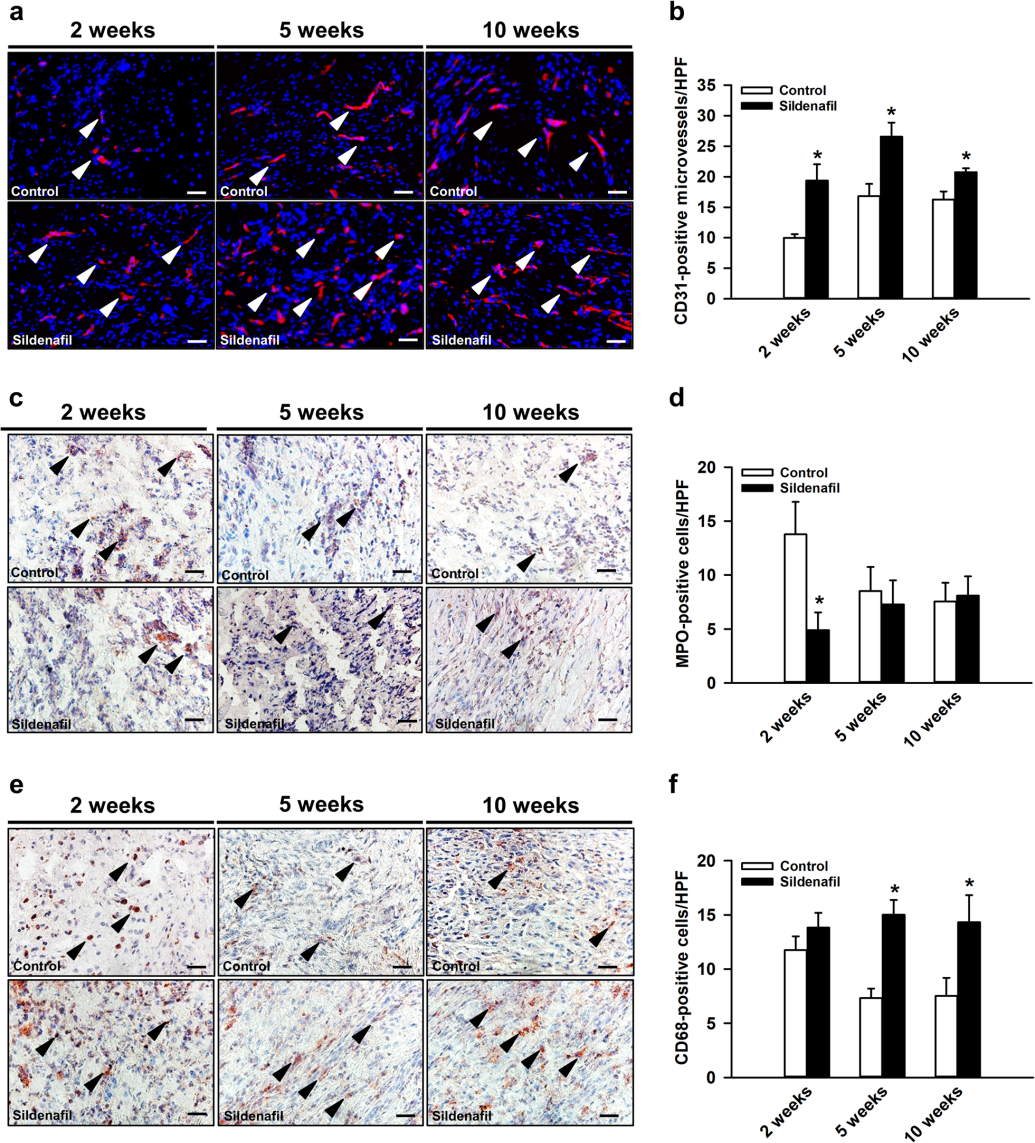
**a** Representative immunofluorescence images of CD31-positive microvessels (arrowheads) within the callus tissue of controls and sildenafil-treated mice at 2, 5 and 10 weeks after surgery. Scale bars: 25 μm. **b** CD31-positive microvessels/HPF within the callus tissue of controls (white bars, n = 8 at 2 and 5 weeks, n = 10 at 10 weeks) and sildenafil-treated animals (black bars, n = 8 at 2 and 5 weeks, n = 10 at 10 weeks) at 2, 5 and 10 weeks after surgery, as assessed by immunofluorescence analysis. **c** Representative immunohistochemical images of MPO-positive cells (arrowheads) within the callus tissue of controls and sildenafil-treated mice at 2, 5 and 10 weeks after surgery. Scale bars: 25 μm. **d** MPO-positive cells/HPF within the callus tissue of controls (white bars, n = 8 at 2 and 5 weeks, n = 10 at 10 weeks) and sildenafil-treated animals (black bars, n = 8 at 2 and 5 weeks, n = 10 at 10 weeks) at 2, 5 and 10 weeks after surgery, as assessed by immunohistochemical analysis. **e** Representative immunohistochemical images of CD68-positive cells (arrowheads) within the callus tissue of controls and sildenafil-treated mice at 2, 5 and 10 weeks after surgery. Scale bars: 25 μm. **f** CD68-positive cells/HPF within the callus tissue of controls (white bars, n = 8 at 2 and 5 weeks, n = 10 at 10 weeks) and sildenafil-treated animals (black bars, n = 8 at 2 and 5 weeks, n = 10 at 10 weeks) at 2, 5 and 10 weeks after surgery, as assessed by immunohistochemical analysis. Mean ± SEM; *p < 0.05 vs. control

The immunohistochemical analysis demonstrated an increased number of CD31-positive microvessels within the callus tissue of sildenafil-treated animals 2, 5 and 10 weeks after surgery when compared to controls (Fig. 7a, b). Moreover, we detected a lower number of MPO-positive granulocytes 2 weeks after surgery under sildenafil treatment (Fig. 7c, d), whereas the number of CD68-positive macrophages was significantly higher within the callus tissue of sildenafil-treated mice at 5 and 10 weeks after surgery when compared to controls (Fig. 7e, f).

### Western blot analysis

**Fig. 8:**
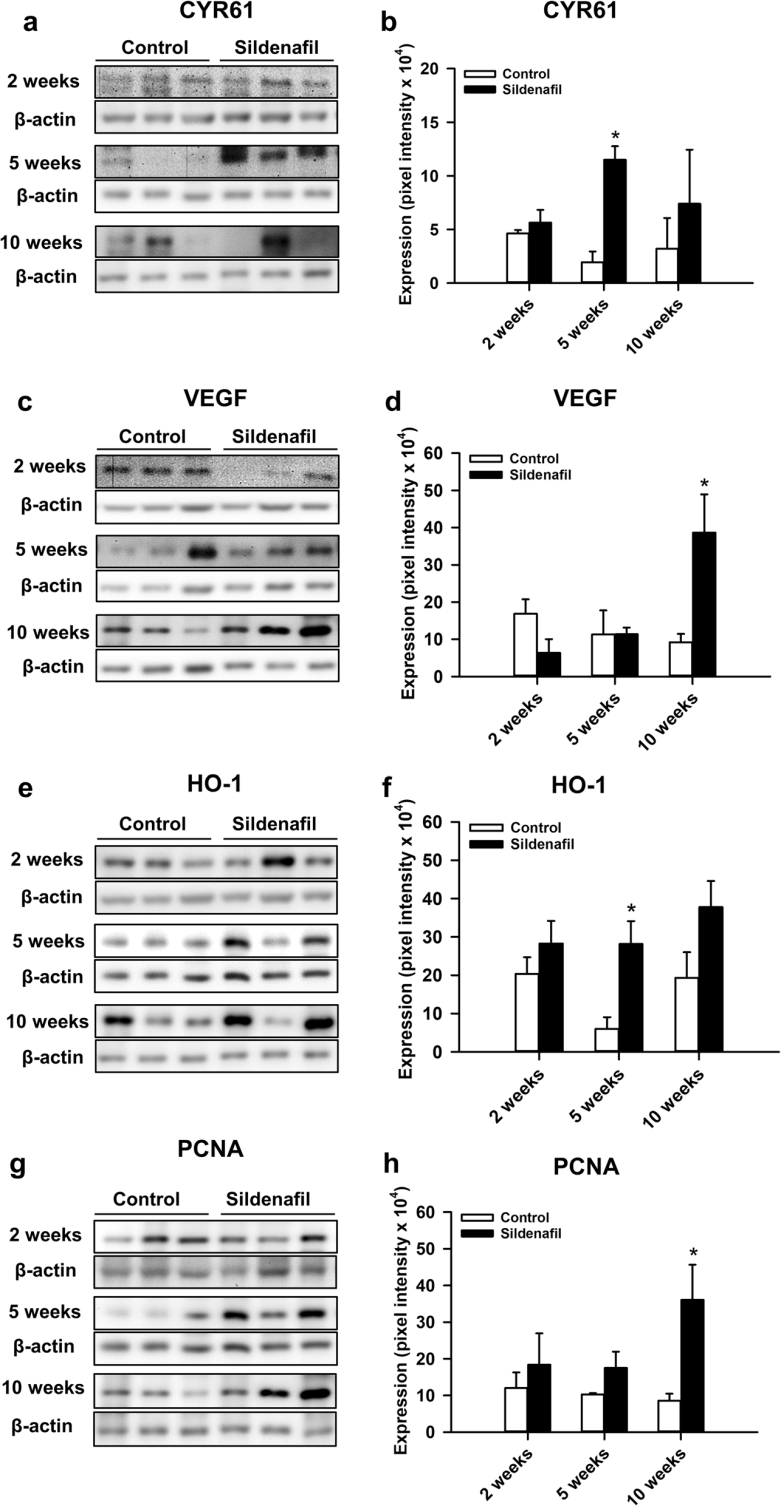
**a** Western blots of CYR61 expression within the callus tissue of controls and sildenafil-treated mice at 2, 5 and 10 weeks after surgery. **b** Expression of CYR61 (pixel intensity ×10^4^) within the callus tissue of controls (white bars, n = 3) and sildenafil-treated mice (black bars, n = 3) at 2, 5 and 10 weeks after surgery. **c** Western blots of VEGF expression within the callus tissue of controls and sildenafil-treated mice at 2, 5 and 10 weeks after surgery. **d** Expression of VEGF (pixel intensity ×10^4^) within the callus tissue of controls (white bars, n = 3) and sildenafil-treated mice (black bars, n = 3) at 2, 5 and 10 weeks after surgery. **e** Western blots of HO-1 expression within the callus tissue of controls and sildenafil-treated mice at 2, 5 and 10 weeks after surgery. **f** Expression of HO-1 (pixel intensity ×10^4^) within the callus tissue of controls (white bars, n = 3) and sildenafil-treated mice (black bars, n = 3) at 2, 5 and 10 weeks after surgery. **g** Western blots of PCNA expression within the callus tissue of controls and sildenafil-treated mice at 2, 5 and 10 weeks after surgery. **h** Expression of PCNA (pixel intensity ×10^4^) within the callus tissue of controls (white bars, n = 3) and sildenafil-treated mice (black bars, n = 3) at 2, 5 and 10 weeks after surgery. Mean ± SEM; *p < 0.05 vs. control

Our Western blot analysis revealed a slightly higher expression of the pro-angiogenic and pro-osteogenic factor CYR61 at 2 and 10 weeks after surgery and an even significantly enhanced expression at 5 weeks after surgery in sildenafil-treated animals when compared to controls (Fig. 8a, b). There was no significant difference in VEGF expression at 2 and 5 weeks after surgery between the two study groups. However, at 10 weeks VEGF expression was significantly higher in sildenafil-treated animals when compared to controls (Fig. 8c, d). The expression of the anti-oxidative and cytoprotective enzyme HO-1 was slightly enhanced at 2 and 10 weeks, and significantly enhanced at 5 weeks after surgery under sildenafil treatment (Fig. 8e, f). In addition, the expression of the proliferation marker PCNA was significantly higher at 10 weeks after surgery in sildenafil-treated animals (Fig. 8g, h).

**Fig. 9:**
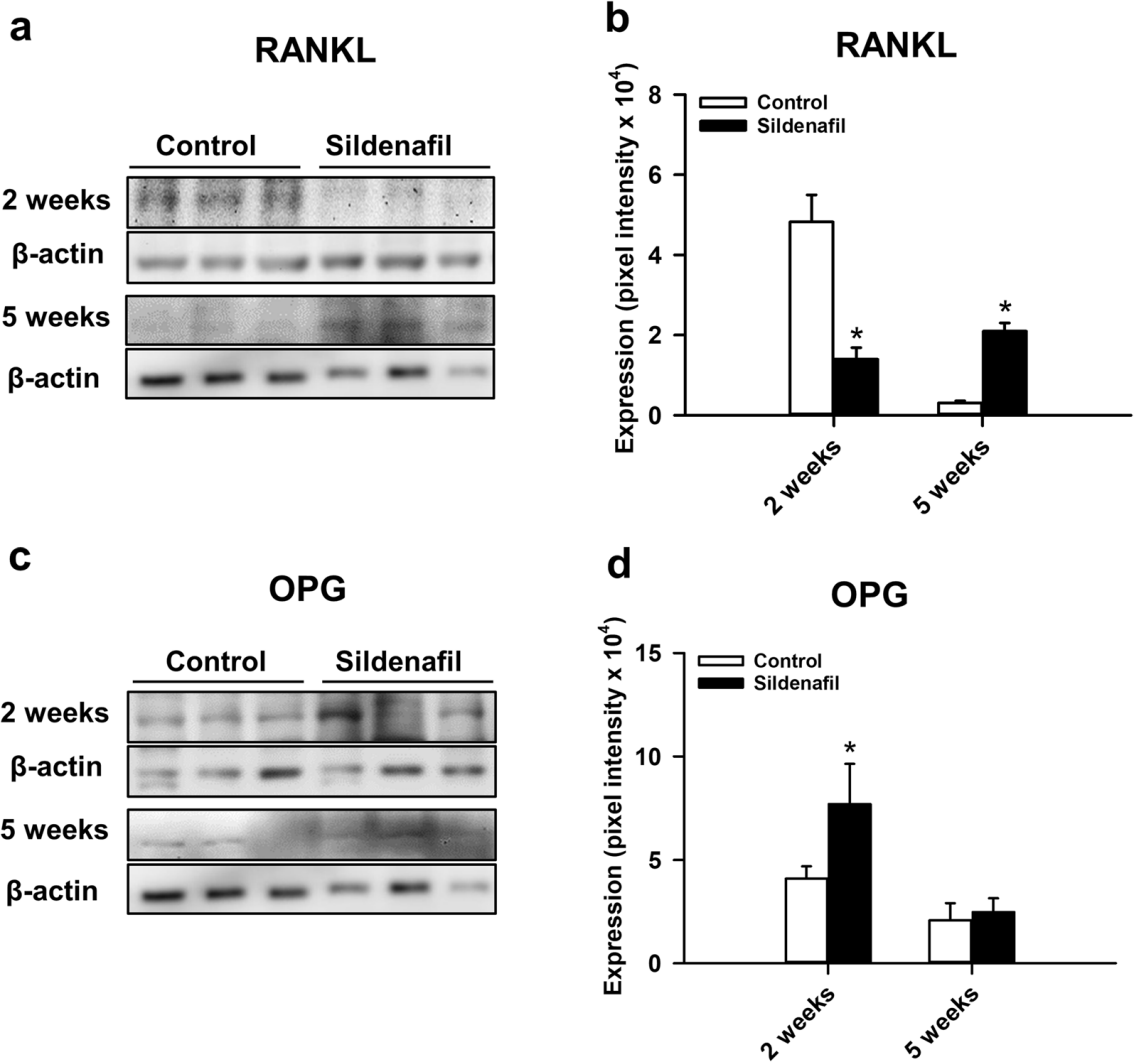
**a** Western blots of RANKL expression within the callus tissue of controls and sildenafil-treated mice at 2 and 5 weeks after surgery. **b** Expression of RANKL (pixel intensity ×10^4^) within the callus tissue of controls (white bars, n = 3) and sildenafil-treated mice (black bars, n = 3) at 2 and 5 weeks after surgery. **c** Western blots of OPG expression within the callus tissue of controls and sildenafil-treated mice at 2 and 5 weeks after surgery. **d** Expression of OPG (pixel intensity ×10^4^) within the callus tissue of controls (white bars, n = 3) and sildenafil-treated mice (black bars, n = 3) at 2 and 5 weeks after surgery. Mean ± SEM; *p < 0.05 vs. control

Moreover, we found that the expression of RANKL, a stimulator of osteoclastogenesis, was significantly lower at 2 weeks after surgery in sildenafil-treated mice, whereas at 5 weeks after surgery the expression of RANKL was significantly higher when compared to controls (Fig. 9a, b). In addition, the expression of OPG, an inhibitor of osteoclastogenesis, was significantly higher at 2 weeks after surgery under sildenafil treatment (Fig. 9c, d).

## Discussion

In the present study, we could demonstrate for the first time that sildenafil stimulates angiogenesis and bone regeneration in an atrophic non-union model in mice. This was associated with a higher bending stiffness of the osteotomized femora, an enhanced oxygen saturation within the callus tissue and an elevated expression of the pro-angiogenic and pro-osteogenic markers CYR61 and VEGF in sildenafil-treated animals.

In our experimental setting, the creation of a 1.8 mm bone defect induced a typical pattern of secondary fracture healing, including endochondral and intramembranous bone regeneration [7, 18]. At 2 and 5 weeks after surgery, our histological analysis revealed that the fracture gap was mainly filled with fibrous tissue in both study groups. All control animals showed no further healing progress at 10 weeks after surgery with only a limited amount of newly formed bone tissue and a complete lack of osseous bridging at the defect site, indicating a reliable non-union formation of 100%. In contrast, sildenafil-treated animals showed a significant progress of bone formation within the osteotomy gap, leading to osseous bridging in 5 out of 10 animals at 10 weeks after surgery.

The improved bone formation under sildenafil treatment was associated with an enhanced formation of microvessels within the callus tissue. This observation is in line with previous experimental studies demonstrating that sildenafil acts angiogenic by upregulating pro-angiogenic factors [15, 16]. Bone is a highly vascularized tissue, which crucially depends on the spatial and temporal interaction of blood vessels and bone cells [28]. Therefore, a sufficient vascularization is also a major prerequisite for successful fracture healing [13, 29]. In fact, newly formed microvessels invade the fracture site and provide oxygen, nutrients and mesenchymal stems cells, which are vital for adequate callus remodeling to woven mineralized bone [30]. A variety of experimental studies could demonstrate that the blockade of angiogenesis by TNP-470, non-steroidal anti-inflammatory drugs and fumagillin results in the inhibition of bone regeneration and eventually non-union formation [31–33]. Reversely, it may be speculated that the herein observed stimulatory effect of sildenafil on bone formation is mediated, at least in part, by its potent pro-angiogenic activity.

Interestingly, our photoacoustic analysis showed a markedly higher oxygen saturation within the callus tissue of sildenafil-treated animals at 5 and 10 weeks after surgery when compared to controls. This finding suggests that the stimulation of angiogenesis by sildenafil results in a continuous supply of the callus tissue with an adequate amount of oxygen. In contrast, we detected a progressive decrease in oxygen saturation within the callus tissue of control mice from 2 to 10 weeks after surgery. This indicates a regressing microvasculature over time, which is not able to supply the surrounding callus tissue with enough oxygen to provide ideal healing conditions throughout the observation period of 10 weeks.

Macrophages play a crucial role in the process of fracture healing and bone regeneration by (i) inducing the migration of mesenchymal stem cells to the fracture site, (ii) promoting collagen I deposition and matrix mineralization and (iii) stimulating anabolic effects on osteogenesis by osteoblast proliferation and differentiation [34, 35]. In fact, Schlundt et al. [36] demonstrated that the depletion of macrophages by clodronate liposomes delays endochondral ossification and hard callus formation. Accordingly, the higher number of macrophages detected in sildenafil-treated animals may have also contributed to the improved bone regeneration.

Moreover, we found a stronger expression of HO-1 under sildenafil treatment. The major function of HO-1 is its defense against reactive oxygen species (ROS) [37]. These toxic radicals are produced during fracture repair and induce cell injury by damaging nuclear acids and proteins as well as causing lipid peroxidation [38]. During fracture repair, the damage induced by ROS is attenuated by antioxidative enzymes such as HO-1, which neutralize free radicals before they can harm cellular components [38]. Therefore, it may be speculated that the higher expression of HO-1 in sildenafil-treated animals facilitates bone regeneration by protecting against the detrimental effects of ROS.

CYR61 has been shown to play a vital role in the process of bone healing by promoting endothelial cell migration and osteoblast proliferation and differentiation [39]. It has been postulated by Lechner et al. [40] that CYR61 acts as an extracellular signaling molecule in human bone due to the immediate early regulation of hCYR61 mRNA by 1α, 25-dihydroxyvitamin D(3) [41]. In addition, Frey et al. [42] reported that CYR61 induces bone formation and callus regeneration, resulting in an enhanced torsional strength in an open fracture model in rabbits. Moreover, we could already demonstrate that sildenafil accelerates fracture healing most probably by a CYR61-induced pathway [17]. In line with these findings, our Western blot analyses showed a significantly higher expression of CYR61 within the callus tissue of sildenafil-treated animals at 5 weeks after surgery when compared to controls. Moreover, the higher expression of CYR61 may influence bone regeneration by acting on chondrocytes and osteocytes. By comparing the course of healing after rigid and semi-rigid fixation in an ovine tibial fracture model, Lienau et al. [43] demonstrated that a prolonged persistence of cartilage is associated with a reduced CYR61 expression. The authors concluded that the reduced expression of CYR61 within the cartilage led to an impaired chondrocyte differentiation and, thus, to a prolonged persistence of cartilage within the callus, ultimately resulting in a delay of healing [43].

VEGF is a central growth factor in the regulation of angiogenesis. Moreover, VEGF plays a major role in the process of bone regeneration [29, 44]. In fact, inhibition of VEGF leads to failed fracture healing and non-union formation in experimental animal models [12, 45]. On the other hand, treatment with VEGF accelerates and improves bone formation [46, 47]. Interestingly, recent experimental studies reported that VEGF does not only stimulate angiogenesis during fracture repair, but also directly induces endochondral and intramembranous bone healing as well as stem cell recruitment and osteoblastogenesis [44, 48, 49]. In the present study, we detected a markedly higher expression of VEGF within the callus tissue of sildenafil-treated animals at 10 weeks after surgery when compared to controls. Thus, it is likely that VEGF contributes to the sildenafil-induced stimulation of bone formation. The late onset of this enhanced VEGF expression may be explained by the fact that the process of endochondral and intramembranous bone healing with subsequent formation of novel bone tissue is delayed in the segmental defect model used in the present study.

Successful bone regeneration requires osteoclast-mediated cartilage and bone resorption within the callus tissue [50]. Accordingly, we found a higher number of TRAP-positive osteoclasts within the callus tissue of sildenafil-treated animals. It is well established that RANKL is a potent stimulator of osteoclastogenesis and osteoclast activity by binding to its corresponding receptor on bone marrow macrophages [30]. OPG, on the other hand, acts as decoy receptor for RANKL, preventing its binding and activation with RANK and, thus, suppressing osteoclast activity [51]. Notably, our Western blot analysis revealed a significantly higher OPG expression in sildenafil-treated animals at 2 weeks after surgery, whereas the expression of RANKL was significantly reduced. At 5 weeks, however, the expression of RANKL was significantly enhanced with no relevant differences in OPG expression. These findings indicate a delayed bone resorption at an early stage of bone regeneration in sildenafil-treated animals caused by a decreased osteoclast activity. During the healing process the osteoclast activity most likely increases, enabling an adequate osteoclast-mediated bone resorption and remodeling. In control mice, the higher osteoclast activity at 2 weeks after surgery most probably leads to an early osteoclastic response, resulting in an impaired process of bone regeneration.

In conclusion, the present study demonstrates that sildenafil stimulates angiogenesis and bone regeneration in a murine atrophic non-union model. Moreover, sildenafil is a clinically approved drug for over 2 decades [52, 53]. This represents a major advantage when compared to novel pharmacological agents, which lack clinical approval and reliable data on potential side effects in humans. Therefore, sildenafil may be a promising compound in the treatment of non-union formation in future clinical practice.

## Data Availability

The datasets during and/or analyzed during the current study available from the corresponding author on reasonable request.
